# Comparison of BTX Profiles and Their Mutagenicity Assessment at Two Sites of Agra, India

**DOI:** 10.1100/2012/272853

**Published:** 2012-04-26

**Authors:** Vyoma Singla, Tripti Pachauri, Aparna Satsangi, K. Maharaj Kumari, Anita Lakhani

**Affiliations:** Department of Chemistry, Faculty of Science, Dayalbagh Educational Institute, Dayalbagh, Agra 282110, India

## Abstract

In the present study, the concentrations of three volatile organic compounds (VOCs), namely, acronym for benzene, toluene, and xylenes (BTX) were assessed because of their role in the tropospheric chemistry. Two representative sites, a roadside and a petrol pump, were chosen for sample collection. VOCs were collected using SKC-activated charcoal tubes and SKC personal sampler and characterized by gas chromatograph using flame ionization detector. Among BTX, benzene had the highest concentration. At the roadside, mean concentration of benzene, toluene, o-,m-xylene, and p-xylene were 14.7 ± 2.4 **μ**gm^−3^, 8.1 ± 1.2 **μ**gm^−3^, 2.1 ± 0.8 **μ**gm^−3^, and 5.1 ± 1.2 **μ**gm^−3^, respectively. At the petrol pump, the mean concentrations of benzene, toluene, o-,m-xylene and p-xylene were 19.5 ± 3.7 **μ**gm^−3^, 12.9 ± 1.1 **μ**gm^−3^, 3.6 ± 0.5 **μ**gm^−3^ and 11.1 ± 1.5 **μ**gm^−3^, respectively, and were numerically higher by a fraction of 2. Monthly variation of BTX showed maximum concentration in winter. Inter-species ratios and inter-species correlation indicated traffic as the major source of BTX. Extracts of samples were positive in both Salmonella typhimurium tester strains TA98 and TA100 without metabolic activation suggesting the presence of direct mutagens in ambient air that can cause both frame-shift and base-pair mutation. The mutagenic response was greater for TA100 than TA98 suggesting greater activity for base-pair mutagenicity than frame-shift mutagenicity and was found to be statistically significant.

## 1. Introduction

Volatile organic compounds (VOCs) comprise a wide range of compounds including aliphatic and aromatic hydrocarbons, alcohols, aldehydes, ketones, esters, and halogenated compounds. The main emission sources of VOCs are evaporative emissions from solvent utilization especially paints and protective coatings and combustion emissions from automobile exhaust [1]. VOCs play an important role in the formation of photochemical smog and tropospheric ozone. They modify the oxidizing capacity of the atmosphere by reacting with hydroxyl radicals (OH) in the presence of NOx to form ozone [2].

Aromatic VOCs in the atmosphere have a high photochemical ozone creation potential, which depends on their structure and reactivity. This has led to the development of scales of “reactivity” or “ozone formation potential” for VOC, of which the most widely used are the “maximum incremental reactivity” scale (MIR), developed by Carter and coworkers [3–5], and the “photochemical ozone creation potential” (POCP) scale, developed by Derwent et al. [6].

VOCs have direct toxic effects from carcinogenesis to neurotoxicity on human beings, and long-term exposure to VOCs is detrimental to human health, for example, by causing sick building syndrome (SBS) [7]. The carcinogenic effects of VOCs are becoming increasingly clear through genotoxicity studies using bacteria [8], mammalian cells [9], animals [10], and epidemiological investigations of large groups of people [11]. Of these assay methods, the salmonella/microsome (Ames) assay is the most widely used, convenient, and effective method to evaluate mutagenicity of airborne particles and source emissions [12].

In urban areas, a group of aromatic VOCs (benzene, toluene, ethylbenzene, and xylene) collectively called as BTEX are of significant concern and constitute up to 60% of nonmethane VOCs [13]. Among BTEX, benzene is an important representative of aromatic hydrocarbons and has been a high-priority urban air pollutant for assessment [14, 15]. It has been reported near heavy road traffic, mobile sources account for 75–85% of the benzene emissions of which 70% is from exhaust. The highest emissions are related to the use of gasoline in noncatalytic cars [16]. Benzene is a well-known human carcinogen for all routes of exposure. According to toxicological studies, Environmental Protection Agency (EPA) has classified benzene as a Group A, human carcinogen, and International Agency for Research on Cancer (IARC) considers benzene as confirmed and probable carcinogen [17, 18]. In addition to being carcinogenic, benzene is also known to be mutagenic while other VOCs are known to have effects on the central nervous system. Toluene is less toxic and causes drowsiness, and impaired coordination. Xylene exhibits neurological effects. High levels from exposure for acute (14 days or less) or chronic periods (more than 1 year) can cause headaches, lack of muscle coordination, dizziness, confusion, and alterations in body balance. Exposure of people to high levels of xylene for short periods can also cause irritation of the skin, eyes, nose, and throat, difficulty in breathing, and other problems with the lungs, delayed reaction time, memory difficulties, stomach discomfort, and possibly adverse effects on the liver and kidneys. Also the reaction of BTEX with hydroxyl radicals and/or nitrate (NO_3_) radicals serves as the dominant degradation processes for aromatic VOCs in the atmosphere [14].

VOCs also contribute to stratospheric ozone depletion and enhancement of the global greenhouse effect [19]. Moreover, the acute and chronic health effects related to VOCs require their monitoring in risk areas.

Knowledge of ambient levels of VOCs is necessary to evolve a proper strategy to control tropospheric ozone build-up and maintain healthy air quality. Inspite of the well-known toxic effects of VOCs, information on VOC levels for Indian cities is limited [15, 20–24]. Aromatic hydrocarbons represent a significant fraction of the volatile organic compounds emitted in urban environment by road traffic [16] and the use of unleaded gasoline which is rich in aromatic hydrocarbons have increased worldwide. Therefore, monitoring of these hydrocarbons in urban area has become an important issue. With this view, the present study aims to report the ambient levels of BTX measured along a roadside carrying heavy vehicular traffic and at a petrol pump from April 2010 to March 2011.

## 2. Methodology

### 2.1. Study Area

Agra is situated at latitude of 27°10′ N and longitude of 78°05′ E with an altitude of 169 m above the sea level in the semiarid zone of India. It is positioned with the Thar Desert of Rajasthan to the west, central hot plains to the south, Gangetic plains to the east, and cooler hilly regions to the north. Agra has a continental type of climate characterized by extreme dryness in summer and cold winters with calm periods. The summer in Agra is hot with intense solar radiation and is dominated by strong southeastern winds (wind speed ranges from 10–16 km h^−1^). Intense solar radiation varies from 19–23 W m^−2^. The winters are associated with calm periods with wind speeds reduced to about 40% (wind speed ranges from 0.5–5 km h^−1^).

**Figure 1:**
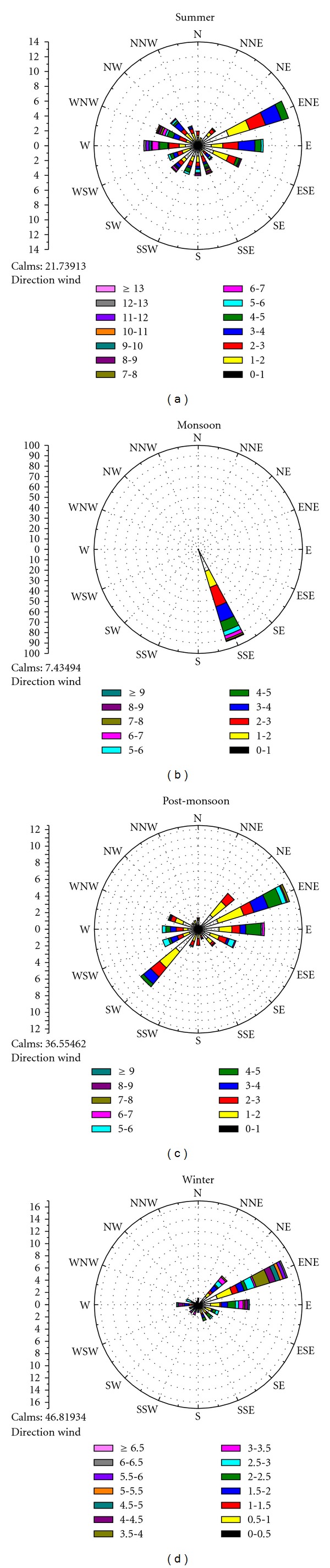
Local prevailing winds at sampling site.

On the basis of rainfall, the study period was divided into four seasons, namely, summer (March–June), monsoon (July-August), post-monsoon (September-October), and winter season (November–February). During the sampling period, meteorological profile measured by automatic weather station (AWS 321) was observed to vary significantly in summer and winter months. In summer months, the average temperature ranged from 35–40°C and average humidity varied from 25–50%. Summers were also associated with strong dust storms (wind speed varied from 10–16 km h^−1^). On the other hand, winters were observed to be associated with calm periods. The average temperature varied from 22–28°C and relative humidity varied from 70–75% in winter months. During monsoon (July–September), relative humidity reached up to 80–85% and average rainfall was observed to be 160 mm.  Table 1 gives the prevailing meteorological conditions during the study period and Figure 1 shows the observed wind direction pattern.

Two sites were selected based on the traffic flow as combustion of fuel specifically gasoline serves as the main source of BTX emissions. Site 1 (roadside) lies in the north of the city and is located along a busy road which caters a mean traffic volume of 10^6^ per day. The site is also surrounded by a stretch of shops of paints and varnishes. Site 2 (A petrol pump) lies about 1 km away from Site 1. These sites were selected to study the variation in emissions of BTX in ambient air and at a point source (petrol pump) as fuel storage and delivery activities serve as a supplementary source to vehicular traffic at petrol pump.

### 2.2. Sampling Period and Sampling Procedure

Ambient air samples were collected during the period of April 2010 to March 2011 along a roadside and a petrol pump. Sampling was carried out by active grab sampling method using a battery-operated pump. A battery, operated portable sampling pump (SKC 224-XR) was used to draw air at the rate of 2 l min^−1^ through SKC adsorption cartridge containing activated charcoal (60–80 mesh). The sampling pump was calibrated by a flow meter SKC making electronic digital flow calibrator. The monitoring schedule followed 4 hourly samples during peak hours, that is, 08 : 00–12 : 00 and 16 : 00–20 : 00 h in a day. On an average, 6 samples were collected every month.

### 2.3. Protection and Storage of Samples

Protection of the exposed charcoal tubes is a very important part of the VOC determination method. Therefore, as soon as the pump was turned off, the tubes were removed from the sampling train and the two open sides were tightly closed using special caps to avoid any desorption and contamination. The sample tubes were put into polythene bags that were tightly closed and were kept in a box in a freezer until processed.

### 2.4. Analysis

Before analysis, all sample tubes were taken from the freezer, contents of the adsorber tubes were emptied into 5 mL tubes containing 2 mL of HPLC-grade carbon disulphide (CS_2_). The tubes were then placed in ultrasonic bath for at least 1 h to obtain the final sample solution. Immediately after this, the microsyringe was washed five times with the sample, washings were discarded successively, and finally 1 *μ*L of aliquot was withdrawn from the samples and injected into a Shimadzu gas chromatograph (GC-17 A) equipped with a flame ionization detector (FID), BP1 capillary column (25 m length and 0.3 mm internal diameter), and GC Solution software. GC oven was programmed for 50°C hold for 4 min and ramped to 250°C at a rate of 10°C/min with 10 min hold at 250°C. Injector and detector temperature was maintained at 250°C. Nitrogen was used as a carrier gas with flow rate of 1 mL min^−1^ and split ratio 1 : 10. The external calibration standard mixture (MISA nonhalogenated volatiles group 17 mix) containing benzene, toluene, ethylbenzene, o-xylene, m-xylene, p-xylene, and styrene, procured from Sigma-Aldrich, was used for the calibration. The samples were quantified against a five-point calibration curve prepared using standards between 2 and 20 *μ*g/mL. Duplicate measurements were done for more than 10% of the samples so as to control the quality of the samples. A blank sample (unexposed tubes) was also run to check for any contamination of the tubes.

### 2.5. Mutagenicity Assay

Mutagenicity assay in the extracts was conducted by the Ames test [25]. In the Ames test, bacteria are mixed with the test substance and then incubated. The number of revertant colonies, called revertants, formed after incubation, serves as an index of activity. Most mutagens and carcinogens identified in air such as certain unsubstituted VOCs require the addition of liver homogenates (referred as S9) to the incubation mixture before they become mutagenic in the Ames test. The liver contains enzymes that metabolically activate mutagens to reactive intermediates. Such compounds are called as indirect-acting mutagens. In this study, two tester strains of Salmonella typhimurium (his^−^) TA100 and TA98 suggested for base-pair mutation and frame shift mutation, respectively, were used. These strains are shown to be most sensitive to mutagens of extract of VOCs [26]. These strains were obtained as lyophilized cultures from Microbial Type Culture Collection and Gene Bank (MTCC), Institute of Microbial Technology (IMTECH), Chandigarh, India.

For performing the Ames test, the extracts of sample were dissolved in 4 mL DMSO, immediately before use. The bacterial strains were initially cultured in culture tubes containing beef extract agar medium, 15–18 h before the experiment. The test was performed in plates containing two layers of agar, the bottom agar (1.5%) providing a suitable support media and the top agar (0.07%) for applying the test chemical and the desired strain. All reagents were prepared as per Maron and Ames [25]. Vogel-Bonner minimal media E (50X) supplemented with agar and glucose was used as bottom agar (25 mL). 2 mL of the top agar consisted of agar, NaCl, and histidine/biotin. The extracts were applied to the plate at dose levels of 1, 2, 4, 8, 16, and 32 *μ*L plate^−1^. In addition to the test plates, a negative control consisting of DMSO solvent blank was used to establish the number of colonies that arise spontaneously for each of the tester strains. Positive control containing known mutagens, dibenzanthracene (DbA) and chrysene (Chy), was also included to confirm the reversion properties and specificities of the strains. DbA and Chy were applied at five dose levels 10, 20, 30, 45, and 60 *μ*g mL^−1^. The plates were incubated at 37°C for 63 hours in dark in inverted position and subsequently the revertant colonies formed in each plate were counted. Counting was performed using a handheld digital colony counter (LA 663, Himedia). Mutagenic response was classified as positive if a reproducible dose-dependent increase of the number of revertant colonies over and above those in the negative control was observed [27]. Mutagenic activity was calculated from the positive slope of the dose-response curve using the regression coefficient *b*, where *y* = *a* + *bx*. Mutagenicity was expressed as number of revertant colonies *μ*L^−1^ of extract. The bacterial background lawn was regularly checked by microscopy, as high doses of the extracts may prove toxic to the tester strains, resulting in a thinning out of background. If massive cell death occurs, the background lawn on the test plates would be sparse compared to control plates.

## 3. Results and Discussion

### 3.1. BTX Concentrations in Ambient Air

The minimum, maximum, and mean concentration of BTX measured at the roadside and petrol pump site is shown in Table 2. Vehicular emissions are reported to be responsible for more than 60% of total VOC emissions [28, 29] and, therefore, traffic seems to be a major source of ambient VOCs in many urban areas. The concentration of BTX at any site would depend upon traffic density and fleet composition. Benzene and toluene with an average value of 14.7 ± 2.4 *μ*g m^−3^ and 8.1 ± 1.2 *μ*g m^−3^, respectively, were observed to be most abundant at roadside. The mean concentration of p-xylene and o-,m-xylene was found to be 5.1 ± 1.2 *μ*g m^−3^ and 2.1 ± 0.8 *μ*g m^−3^, respectively. Similar variation was also observed at petrol pump but the levels were elevated.

**Figure 2:**
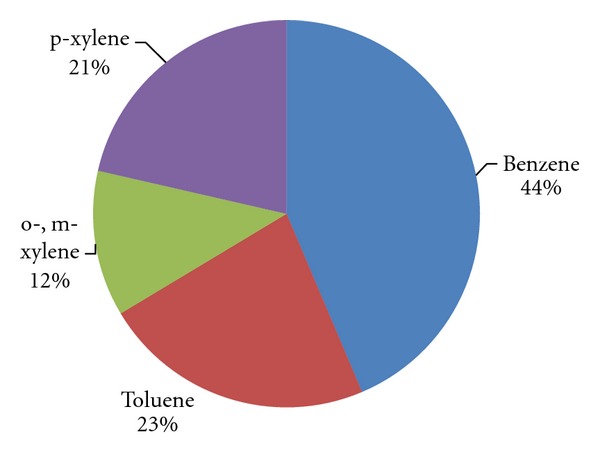
Percent abundance of VOCs at roadside.

The higher concentration of benzene and toluene might be probably due to their emission from gasoline (major fuel of vehicles) which is known to contain high proportion of these compounds, their longer lifetime, and lesser reactivity with OH radical as compared to xylenes (shorter lifetime and higher reactivity).  Figure 2 shows the percent abundance of observed BTX. It is observed that benzene (44%) and toluene (23%) contribute to more than 50% of total VOCs (sum of benzene, toluene, o-,m-xylene, and p-xylene) emitted.

Benzene and toluene with atmospheric lifetimes of 12.5 and 2 days, respectively, are known to be relatively stable and do not dissipate into the environment immediately after release. Xylene, however, has a lifetime of 7.8 h only and usually does not remain long in the atmosphere [15]. Further, benzene and toluene being monoaromatic hydrocarbons react very slowly with O_3_ and NO_3_ radicals, their rate constants being in the order of <10^−20^ and 10^−16^ cm^3^ mole^−1^ s^−1^ [30]. Thus their depletion in the atmosphere via these reactions is negligible. The only significant process for their atmospheric loss is their photochemical reaction with OH radicals, having rate constants 1.23 × 10^−16^ and 5.96 × 10^−12^ cm^3^ mol^−1^ s^−1^, respectively [30].

**Scheme 1:**
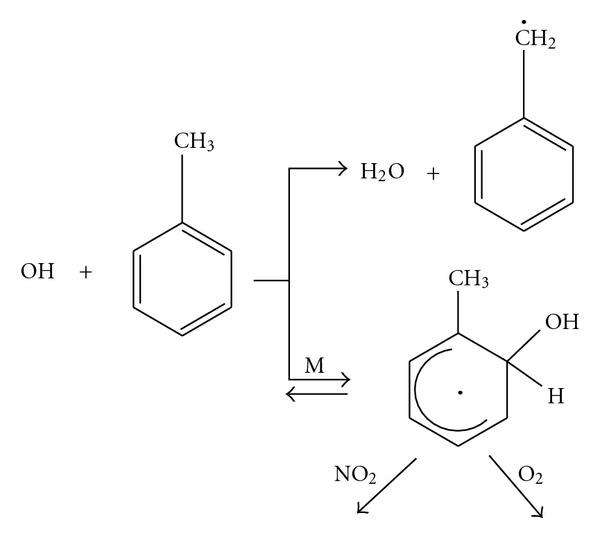


As evident from temperature-dependent kinetic studies, the OH radical reactions proceed by two pathways; H-atom abstraction from the C–H bonds of the alkyl substituent groups, and addition of the OH radical to the aromatic ring as shown in Scheme 1.

**Figure 3:**
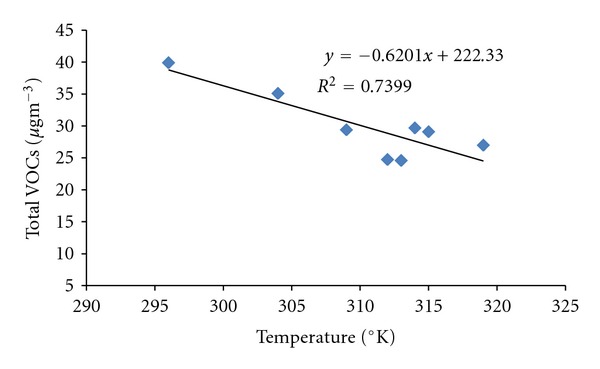
Benzene concentration (monthly mean) plotted as a function of temperature.

The photochemical reactions with OH radicals are known to have temperature dependence. During the study period (April 2010 to March 2011), the temperature varied from approximately 300–320°K in summer months and 285–300°K in winter months. Based on temperature conditions, it is expected that the removal of BTX would have probably taken place through abstraction of H-atom mechanism in summer months which proceeds under higher temperature conditions. Therefore, comparatively lower levels of BTX were observed in summer months than winter months. The annual variation of VOCs is better reflected if the concentration is plotted as a function of temperature observing a significant negative correlation between benzene concentration and temperature as observed in previous studies [31, 32]. As expected, considerable negative correlation (*R* ^2^ = 0.73) was observed between benzene concentration and temperature as shown in Figure 3. Toluene and xylenes are largely used as solvents and, therefore, their existence may come from sources other than traffic too. In addition, photochemical degradation of toluene and xylenes is faster than that of benzene [31], implying that it is easier to see the relationship between benzene and traffic density.

**Figure 4:**
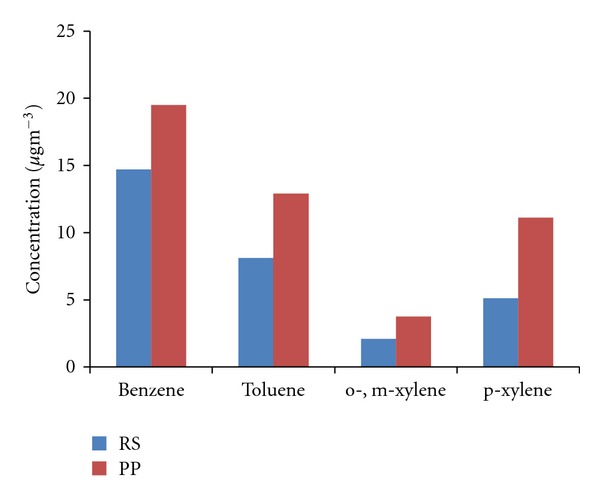
Comparison of BTX at roadside and petrol pump.

Figure 4 shows the comparison of VOCs observed at two sites. It is observed that comparatively higher concentrations of benzene and toluene were observed at petrol pump than roadside measurements. The concentration of Benzene varied from 9.3 *μ*g m^−3^  to 25.8 *μ*g m^−3^, Toluene varied from 2.1 *μ*g m^−3^ to 25.2 *μ*g m^−3^, o-,m-xylenes varied from 4.8 *μ*g m^−3^ to 9.9 *μ*g m^−3^, and p-xylene varied from 4.8 *μ*g m^−3^ to 13.2 *μ*g m^−3^ at petrol pump. The concentration levels were found to be comparable with the levels reported at filling stations in France [33] and Greece [34]. It can be concluded that the air at the petrol pump does not have the same proportion of VOCs as the general city air. Presumably, the VOC concentrations at petrol pump are influenced by both moving vehicular traffic and the petrol pump activities. These activities include fuel storage and delivery, filling, and emptying activities of tanks containing petrol causing displacement losses. As we go further from petrol pump, the VOC concentrations are closer to that found in the general air.

The independent samples *t*-test was applied to further confirm whether the concentrations of benzene, toluene, o-,m-xylene, and p-xylene along the roadside differ significantly from the concentrations observed at petrol pump site. The null hypothesis indicates that concentrations of benzene, toluene, o-,m-xylene, and p-xylene along the roadside and petrol pump are similar. On applying *t*-test, *t*-value and *P*-value were found to be 2.63 and 0.018 for benzene, 3.42 and 0.004 for toluene, 1.55 and 0.146 for o-,m-xylene, and 2.34 and 0.063 for p-xylene. On comparing calculated *t*-value and *P*-value with the tabulated value, it was found that null hypothesis is rejected at 5% significance level for benzene and toluene and accepted for o-,m-xylene and p-xylene. Hence, *t*-test is found to be statistically significant with the 95% significance level for benzene and toluene, that is, the concentration of benzene and toluene along the roadside differs significantly from the concentration at petrol pump. This might be probably because benzene and toluene are constituents of gasoline and are emitted into the atmosphere by car exhausts [20]. Although the concentrations are numerically higher at petrol pump site, *t*-test indicates variation at two sites is not statistically significant for o-,m-xylene and p-xylene.

### 3.2. Comparison with Other Sites

Table 3 presents the comparison of Benzene and Toluene levels at Agra with other sites. The levels of Benzene and Toluene were observed to be comparable to Northern Germany [31], Mexico [35], Naples [36], and Barelona City [32] and much lower than other megacities like Mumbai [24], Delhi [21], China [37], Taiwan [38], Hyderabad [39], Zabrze [40], and Kolkata [41]. Comparatively higher concentrations of Benzene and Toluene might be due to more traffic volume and hence greater exhaust emissions.

### 3.3. Monthly Variation of BTX

**Figure 5:**
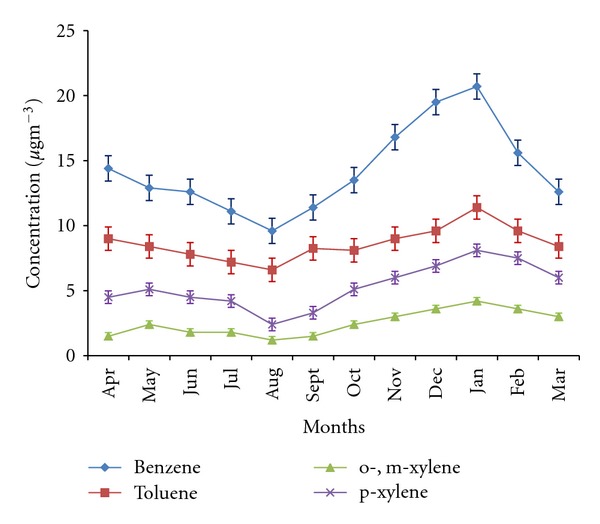
Monthly variation of BTX.

Figure 5 shows the monthly variations of the concentrations of the BTX measured at this site. Benzene was found to be the most abundant component followed by Toluene. BTX shows the expected summer minimum and winter maximum concentrations. Benzene, toluene, o-,m-xylene, and p-xylene varied from its maximum concentration 20.7 *μ*g m^−3^, 11.4 *μ*g m^−3^, 4.2 *μ*g m^−3^, 8.1 *μ*g m^−3^ in winter months, respectively, to as low as 9.6 *μ*g m^−3^, 6.6 *μ*g m^−3^, 1.2 *μ*g m^−3^, 2.4 *μ*g m^−3^ in summer months, respectively. Monthly variations are primarily dependent on emission patterns, meteorology, variations in the source strength, and most essentially photochemistry between VOCs and OH that results in the removal of VOC species from the atmosphere [42].

Agra has a continental type of climate with dry summers and winters associated with calm periods occurring for almost 40% of the time. Although traffic emissions are expected to remain reasonably constant throughout the year, the higher winter values might be probably due to calm conditions and stability of the atmosphere wherein dispersion and dilution of pollutants is restricted due to temperature inversion phenomenon and low mixing heights. Higher levels of BTX have also been reported by Hoque et al. [20] at Delhi. On the other hand, summer experiences unstable atmosphere associated with high solar radiation, high temperature, and frequent occasions of dust storms locally known as *andhi*. The high solar radiation acts as a catalyst for the easy dissociation of species like ozone, aldehydes, and so forth, leading to formation of OH radical. The higher concentration of OH radical and frequent dust storms in summer plays a key role in enhanced mixing and rapid dissipation of pollutants resulting in their lower levels in the atmosphere through atmospheric clean up and degradation of VOCs. Hakola et al. [43] have reported higher OH radical concentration in summer months as compared to winter months in Central Finland.

### 3.4. Interspecies Ratios

Interspecies ratios of the aromatic VOCs were used as an indicator to compare the BTX emission sources. The ratios of VOCs with varying reaction rates against OH provide information about the characteristics of air at the sampling site. Interspecies ratio is listed in Table 4.

Toluene-to-Benzene (T/B) ratio has been commonly used as an indicator of traffic emissions. Benzene and Toluene are chief constituents of gasoline emitted into the environment by vehicular exhausts. Lee et al. [13] suggested that T/B ratio increases with increasing traffic volume, industrial emissions, and other urban sources. In the present study, value of T/B ratio was observed to be 0.74. The (T/B) ratios found in the present study were comparable with those observed in Thane (0.61; [24]) and Southern Taiwan (0.2–4.6; [44]) and lower than those found in Rome (2.8), Izmir (1.87–2), and Copenhagen (2.2) [14, 45, 46]. The difference of (T/B) ratio among these cities is due to differences in vehicle types, fuel composition, and industrial activities.

The lower values of (X/B) ratio (0.26) indicate higher Benzene concentration and suggest that more reactive species have been exposed to photochemical degradation. This lower (X/B) ratio also indicates aging of air mass. The ratio at this site is observed to be higher than ratio observed at Thane (0.06, [24]) and lower than China (0.71, [47]).

### 3.5. Interspecies Correlation

Several work [14, 36, 48] have used correlation analysis to elucidate the possible sources of the aromatic VOCs. A good mutual correlation among the species indicates that they might primarily originate from the same source.

In the present study, Pearson's correlation (2-tailed) of concentration of BTX was calculated (Table 5). A good mutual correlation between o-,m-xylene and benzene (*r* = 0.9) indicates that they might possibly originate from gasoline sources [15, 49]. Since benzene mainly comes from traffic source, it can be used as an indicator of other aromatic compounds in heavy traffic areas; but benzene shows lower “*r*” values with toluene and p-xylene thereby indicating spiking of these VOCs from some additional source apart from vehicular source. The additional VOC source might be a chain of shops of paints and varnishes at this site.

### 3.6. Ranking of BTX with respect to Ozone Formation Potential

The ranking of airborne pollutants on a mass concentration (*μ*g m^−3^) basis is of interest in order to assess human exposure to toxic compounds like benzene, it is also of interest to examine the relative importance of these pollutants for their role in photochemical smog formation [3] including production of ozone [4].

Generally, the maximum incremental reactivity (MIR) is popular in the assessment of ozone formation potential of various VOC compounds [50]. Carter's MIR is the amount (in grams) of ozone formed per gram of VOC added to an initial VOC-NOx mixture, indicating how much a compound may contribute to ozone formation in the air mass [4]. These unitless MIR coefficients are intended for use in relatively high NOx conditions, which may be used as an important tool in ozone control programs. The reactivity of VOC with OH radical depicts the ability of the hydrocarbon to form higher oxidized products like aldehydes, ketones, acids, organic peroxy radicals, and so forth.

The ranking of the BTX species according to mass concentration, ozone formation potential, and reaction with OH is given in Table 6. The rate constants of VOC-OH reactions and MIR coefficients were taken from the literature [3, 4, 51]. The concentration of benzene was found to be maximum (14.7 *μ*g m^−3^) followed by toluene (8.1 *μ*g m^−3^), p-xylene (5.1 *μ*g m^−3^), and o-,m-xylene (2.1 *μ*g m^−3^). Although the concentration of xylenes were observed to be minimum among the BTX, they were found to be the most dominant contributor to ozone formation based on the MIR scale and reaction of VOCs with OH. The results obtained are found to be in agreement with the observations of McCann and Ames [52] at Seoul and Grosjean et al. [53] at Porto Alegre. Benzene being the most abundant and hazardous species among BTX showed minimum potential to ozone formation. Toluene was found to have the second largest contribution to ozone formation.

### 3.7. Mutagenicity of VOCs

**Figure 6:**
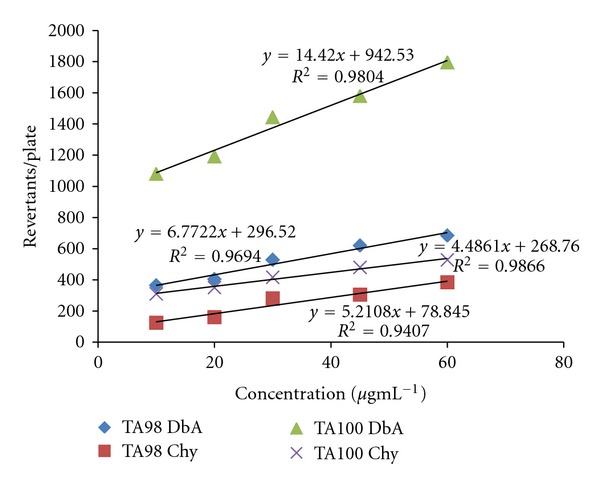
Dose-response curves for standard mutagens (DbA and Chy).

A number of environmental chemicals have been detected as mutagens in *in vitro* tests and have subsequently been identified as animal carcinogens. It is likely that environmental factors initiate most human cancer, and it is becoming increasingly apparent that the causative agents in these environmental factors are likely to act by damaging DNA, for example, cigarette smoke, asbestos, and known human chemical carcinogens [54]. With this view, Salmonella typhimurium test strains TA98 and TA100 were exposed to VOC samples without the addition of metabolizing enzymes (S9). Therefore, only direct acting mutagens were detected. Simultaneously, negative control of DMSO and positive control of known mutagens (DbA and Chy) were also tested. According to the criteria given by Maron and Ames [25], results were considered positive if the number of revertants on the plates containing the test concentrations gets double with the spontaneous reversion rate (with respect to negative control) and a reproducible dose-response relationship is obtained. The dose-response curves were obtained by regression plot of dose versus number of revertant colonies (Figure 6). The mutagenic activity of the extracts, expressed as the number of revertants *μ*L^−1^ of extract was calculated from the slope of the linear dose-response curve: *y* = *a* + *bx*, where *y* is the number of revertants plate^−1^, *x* is the concentration of the extract (*μ*L plate^−1^), *a* is the intercept, and *b* is the slope of the regression line (the number of revertants *μ*L^−1^).

Positive results were found for both the standard compounds (DbA and Chy) while the negative controls showed insignificant growth of colonies. From the slope of the regression line (Figure 6), it is evident that the mutagenic activity of DbA is 2-3 folds greater than Chy in both the strains, suggesting its greater carcinogenic potency as compared to Chy. This finding is also consistent with the order of potential carcinogencity and bioactivity of PAH [55].

**Figure 7:**
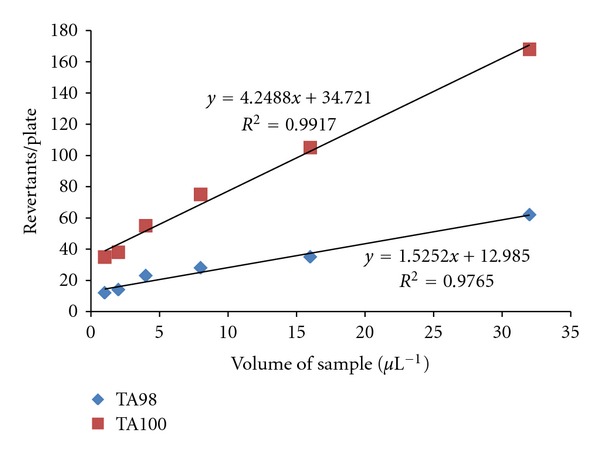
Dose-response curves for VOC extracts.

Figure 7 shows the linear dose-response curves of VOC samples exposed to the two bacterial strains TA98 and TA100 within the range of the selected concentrations, that is, 1–32 *μ*L^−1^. The number of revertants plate^−1^ increased linearly for TA98 strain TA100 strain. Hence, significant increase in the number of revertants plate^−1^ was observed. From the slope of regression line, it is observed that extracts of these samples induced and increased number of revertant colonies linearly with the increase of dose level. This linear increase is an indication of presence of both frame shift mutagens as well as mutagens capable of causing base pair substitutions. Further, the higher slope value of TA100 (*m* = 4.248) than TA98 (*m* = 1.525) indicated greater mutagenic activity of extract toward TA100 strain and, therefore, it was concluded that the extracts had a higher potential for base pair mutagenicity as compared to frame shift mutagenicity.

Several other researchers have also reported higher revertants plate^−1^ for TA100 in contrast to TA98 for organic extracts from diesel exhaust particles [56], biofuel combustion [57], and photocatalytic degradation of toluene [58]. Similar studies on mutagenicity assays have been conducted on exhaust from mobile sources like natural gas fueled truck [59], diesel passenger cars [60], and motorcycles exhaust [61]. The higher mutagenicity of TA100 strain than TA98 strain was further validated by applying independent *t*-test. The null hypothesis indicates that the numbers of revertant colonies obtained using TA98 and TA100 strain are similar. The *t*-value and *P*-value were found to be 2.291 and 0.035, respectively. On comparing calculated *t*-value and *P*-value with the tabulated values, *t*-test is found to be significant with 95% significance level, and the null hypothesis is rejected at 5% significant level. Here, it is further concluded that the numbers of revertant colonies obtained using TA98 and TA100 strains are different.

## 4. Conclusion

Analysis of ambient air samples at two sites, roadside and petrol pump indicated that BTX concentration levels are mainly, influenced by the road traffic. The mean concentrations of benzene, toluene, o-,m-xylene, and p-xylene were 4.9 ± 0.4 *μ*g m^−3^, 2.7 ± 0.8 *μ*g m^−3^, 0.69 ± 0.2 *μ*g m^−3^, and 1.7 ± 0.2 *μ*g m^−3^, respectively, at roadside and the mean concentrations of benzene, toluene, o-,m-xylene, and p-xylene mean concentrations were 6.5 ± 1.7 *μ*g m^−3^, 4.3 ± 1.1 *μ*g m^−3^, 1.2 ± 0.5 *μ*g m^−3^, and 3.7 ± 0.5 *μ*g m^−3^, respectively, at petrol pump. The higher BTX concentrations at petrol pump were found to be statistically significant at 95% significance level and are presumably due to contribution from both moving vehicular traffic and petrol pump activities. BTX levels were higher in winter than summer. The toluene/benzene ratio (0.74) and xylenes/benzene (0.26) ratios reflected the emission of road traffic and ageing of air mass, respectively. Interspecies correlation indicates the possible origin of BTX from gasoline sources. The extracts of samples were mutagenic. TA100 strains showed a greater mutagenic response indicating higher potential for base pair mutagenicity compared to frame shift mutagenicity and independent *t*-test was significant at 95% significance level.

## Figures and Tables

**Table 1:** Range of temperature, relative humidity, and wind speed observed during the study period.

Sampling period	Temperature (°C)	Humidity (%)	Wind speed (km h^−1^)
April'10	17–45	4–27	5–12.5
May'10	23–46	4–45	9–24
June'10	25–48	12–63	4–19
July'10	24–40	41–94	3–17
August'10	22–38	45–100	3–12
September'10	19–39	56–100	1–14
October'10	12–36	55–100	1–17
November'10	7–31	48–100	1–13
December'10	3–25	84–100	1–3
January'11	2–24	53–100	1–4
February'11	5–29	57–100	1–7
March'11	8–37	11–66	2–12

**Table 2:** Concentrations of BTX (*μ*g m^−3^) at roadside and petrol pump.

Hydrocarbon	Roadside (*N* = 74)			Petrol pump (*N* = 50)		
	Minimum	Maximum	Mean ± standard deviation	Minimum	Maximum	Mean ± standard deviation
Benzene	5.1	20.4	14.7 ± 2.4	9.3	25.8	19.5 ± 3.7
Toluene	2.7	9.9	8.1 ± 1.2	2.1	25.2	12.9 ± 1.1
o-,m-xylene	1.5	3.6	2.1 ± 0.8	4.8	9.9	3.6 ± 0.5
p-xylene	3.9	6.9	5.1 ± 1.2	4.8	13.2	11.1 ± 1.5

**Table 3:** Comparison of benzene and toluene with other sites.

	Benzene (*μ*g m^−3^)	Toluene (*μ*g m^−3^)	Reference
Mumbai	13.4–38.6	10.9–33.5	[24]
Hannover (Northern Germany)	9.6	25.7	[31]
China	15.4–67.3	28.6–106.9	[37]
Mexico City Metropolitan Zone	5.29	28.22	[35]
Kaohsing City Site 1	10.97	43.36	[38]
Taiwan Site 2	13.28	54.49	
Delhi	174.7–369.4	—	[21]
Zabrze Aug-Sep 2001	0.3–145.4	0.4–100.7	[40]
Aug-Sep 2005	0.3–113.7	0.4–200.6	
Barcelona City Rural	0.2–8.3	0.5–95.4	[32]
Urban	0.5–12.4	4.3–121.3	
Naples Metropolitan Area	4.4–17.2	15.8–57.7	[36]
Near Suburban Area	3.6–11.8	8.1–27.3	
Far Suburban Area	2.3–12.8	5.6–30.3	
Hyderabad	120–173	110–126	[39]
Kolkata	13.8–72.0	21.0–83.2	[41]
Agra	14.7 ± 2.4	8.1 ± 1.2	Present study

**Table 4:** Interspecies ratio.

	Ratio
Toluene/benzene	0.74
Xylene/benzene	0.26

**Table 5:** Interspecies correlation.

	Benzene	Toluene	o-,m-xylene	p-xylene
Benzene	1			
Toluene	0.7	1		
o-,m-xylene	0.9	0.6	1	
p-xylene	0.5	0.5	0.4	1

**Table 6:** MIR coefficient, VOC-OH rate constant, and ranking of BTX according to mass concentration, ozone formation potential, and reaction with OH leading to formation of oxidants.

Hydrocarbon	MIR coefficient	OH*	VOC (*μ*g m^−3^)	O_3_ formation potential^a^	Reaction with OH^b^
Benzene	0.42	1.23	14.7	6.2	1.8
Toluene	2.7	5.96	8.1	21.9	12.6
o-,m-xylene	8.2	23.6	2.1	16.9	11.1
p-xylene	6.5	13.7	5.1	33.2	15.8

^a^VOC × MIR.

^b^VOC in ppb × VOC-OH* rate const. (10−12 cm^3^/molecule/s) multiplied by 10^12^.
